# A Scoping Review of Pain Management Education Programs (PMEPs): Do They Prepare Nurses to Deal with Patients' Postoperative Pain?

**DOI:** 10.1155/2020/4062493

**Published:** 2020-10-15

**Authors:** Manaporn Chatchumni, Henrik Eriksson, Monir Mazaheri

**Affiliations:** ^1^School of Nursing, Rangsit University, Pathumthani, Thailand; ^2^Department of Health Sciences, The Swedish Red Cross University College, Stockholm, Sweden; ^3^Department of Health Sciences, University West, Trollhättan, Sweden

## Abstract

This is a report of a scoping review undertaken to obtain an overview of studies conducted on pain management education programs (PMEPs). The aim of this review was to describe existing research publications relating to PMEP to map how pain management practice training might directly influence surgical nurses in contributing to successful pain outcomes in patients. The initial search of electronic databases identified 40 articles according to the inclusion criteria and search strategy, which applied the following terms: (“Pain management education program”) AND^*∗*^ OR^*∗*^ (“Nurses”) AND^*∗*^ OR^*∗*^ (“Patient outcomes”) AND^*∗*^ (“Mixed methods”). Titles, abstracts, and keywords were also searched for the term “Nurse education.” After applying exclusion criteria, five relevant peer-reviewed articles were eventually selected for the final charting of the data. The search included articles published between January 2015 and March 2019. The results show that PMEPs employ a variety of computer-based simulation, web-based facilitation, and video materials based on an evidence-based approach in their syllabuses. PMEPs were shown to enhance practice by promoting improved skills in critical thinking, leadership, patient management, and health promotion. Additionally, these programs promote an ability to practice across a variety of inpatient and outpatient settings, wherein nurses' engagement in managing patients' pain increased after completing the PMEP. Research within PMEP indicates that these programs may contribute to promoting opportunities for new collaborations within multidisciplinary team projects. Additionally, further research initiatives are needed to explore various aspects of these programs to enhance the nursing skills required for effective pain management, such as computer-based simulation, web-based facilitation, and video materials. Moreover, research relating to PMEPs in low- and middle-income countries is scarce and warrants further study.

## 1. Introduction

Pain management education programs (PMEPs) are limited to only one hour of teaching within the nursing curricula at most schools offering bachelor's degree qualifications [1, 2]. Student nurses and newly registered nurses often have insufficient nursing practice experience and training to optimally care for patients affected by perioperative pain. The literature does paint a realistic picture of nursing pain management practices, revealing how best to manage the pain of their patients [3–5]. However, much effort is still needed to improve the effectiveness of pain management practices. Furthermore, nurses' pain management practices are complex and involve a variety of different management structures that are interconnected and codependent on each other. Previous research illustrates that pain management does not follow one standard set of practices; it differs between healthcare professionals and includes several complex structures within which nurses must navigate in managing the patient's pain [6]. It has been suggested that management structures influence nurses' practices, and, when responding to and addressing patients' postoperative pain, these reside within a complex model consisting of nursing culture in assessing pain scores and managing pain; nurses' responses to patients in pain, and nurses' own experiences of assessing and managing pain [6].

Despite improvements in nursing guidelines and instructions in managing patients' pain, some studies indicate that, from the patient's perspective, patients often attempt to conceal their symptoms and are thus exposed to even worse pain [7], suggesting that healthcare professionals must understand the cultural context and how this might influence their clinical practices in order to manage their patients' pain effectively [4, 5]. Uncontrolled pain has been shown to delay the patient's recovery after surgery, which can lead to an adverse effect on aspects of quality of life, including physical, psychological, social, and economic factors, and which has been identified as a significant gap in the knowledge in relation to clinical nursing education programs [2, 7]. Providing sufficient pain relief is associated with favorable clinical outcomes and improved cost-effectiveness in healthcare systems. As such, nurses play a major role in pain management practices that involve continuity of care in controlling patients' pain [8], and nurses' systems for responding to a patient in pain are an important concern in the field of pain management. A systematic review found that the assessment of pain by physicians and nurses showed moderate to good levels of assessing pain accuracy when compared with the patients' own estimations of pain intensity [9]. Additionally, emerging evidence recommends that nursing students are trained for competency in effective pain management practices and that training programs for qualified nurses take into consideration the poor levels of pain assessment accuracy observed in providers who have limited clinical experience (<4 years) [7–10].

Nurses need improved knowledge regarding the impact that educational strategies have on patients' complications from pain symptoms. Thus, an evaluation of educational interventions for effective pain management is needed. This study comprised the performing of scoping review of the literature to obtain empirical information relating to nurse education and practice and also to provide an understanding of how to shape skill acquisition in the transition process between being a nursing student, with limited clinical experience, and becoming a registered nurse [8]. The aim of this study is therefore to describe existing research publications relating to how pain management education programs (PMEPs) influence the pain management practices of surgical nurses, with an emphasis on how such training can lead to more successful patient pain outcomes.

## 2. Material and Methods

### 2.1. Methods

This study adopted a scoping review design, conducted by searching the literature in five steps, as recommended by Arksey and O'Malley [11], as follows: (1) identify the research questions, (2) identify the relevant studies, (3) select the studies that are most relevant, (4) chart the data, and (5) summarize and present the results. A scoping review design is appropriate when the goal is to summarize the research findings relating to a range of research in a particular area of study that has not been comprehensively reviewed before [12]. Further, Mays et al. [13] explain that the aim of a scoping review is to map the key concepts that underpin a research area and to present the available evidence for this area. This study completed the review in five steps, as follows

**Figure 1:**
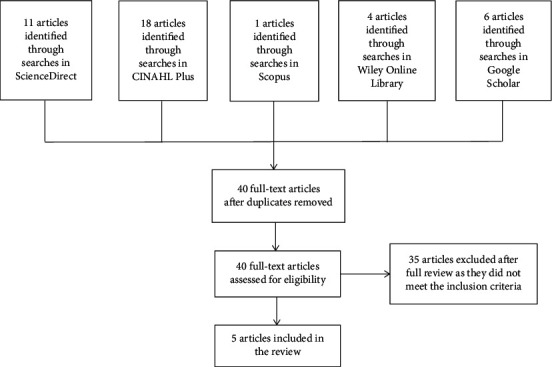
The process from identification to inclusion of articles in the review.

  First, the research questions were identified: What is the impact of pain management education programs (PMEPs) on surgical nurses, and what is the effectiveness of PMEP for improving patient outcomes, when evaluated with mixed-methods approaches?
  Second, relevant publications were identified by searching through several electronic databases, including ScienceDirect, CINAHL Plus, Scopus, Wiley Online Library, and Google Scholar. The search strategy employed the terms as follows: (“Pain management education program”) AND^*∗*^ OR^*∗*^ (“Nurses”) AND^*∗*^ OR^*∗*^ (“Patient outcomes”) AND^*∗*^ (“Mixed methods”); as well as searching the title, abstract, and author-specified keyword for the term “Nurse education.” The inclusion criteria for type of literature were journal articles, conference proceedings, and monographs published between January 2015 and March 2019, and which were written in English. We retrieved a total of 40 publications from searches of the following databases: ScienceDirect, 11 papers, including abstracts and full papers; CINAHL Plus, 1 full paper; Scopus, 18 full papers; Wiley Online Library, 4 full papers; and Google Scholar, 6 full papers. A double-search operation was performed by two authors, each of whom recorded their own results and screened and checked those of the others to verify that the titles, abstracts, and keywords adhered to the inclusion criteria.
  Third, we performed a further selection of the identified studies by sifting and sorting the articles in accordance with the inclusion criteria: mixed-methods research, education programs related to nursing care in postoperative pain management, and postqualified training programs with groups of nurses. Each article was reviewed in relation to the study population, aims, methods, outcomes, and the reliability of the results. Additionally, we verified whether each article reported having sought ethical approval as part of this quality assessment, and a scoping review was appropriate regarding the pain management education programs in terms of the volume, nature, and characteristics of the empirical research in the field and to identify any gaps. After checking for duplicates, a total of 40 articles were identified. Of these studies, 24 were excluded due to insufficient study design; 10 did not evaluate a pain management education program; and one did not evaluate an intervention of interest. After this initial appraisal of the identified publications to assess their “best fit” to the inclusion criteria of our scoping review, a final total of 5 articles were included in this study (Figure 1), which are displayed in the data matrix included in Table 1. The excluded studies and the reasons for their exclusion are shown in Table 2.
  Fourth, we charted the data by mapping key items of information obtained from the review of the primary search, as shown in Table 1.
  Fifth, the results were described in relation to the impact of PMEP on the practices of surgical nurses and the effectiveness of PMEP on patient outcomes, based on research employing a mixed method as the research design. A summary of the study characteristics of each publication included in the literature review is presented in Table 2.

## 3. Results

All of the studies were conducted in the United States of America (USA); however, the study settings varied. One publication presented the results of a pilot study and included 30 nursing students attending a large metropolitan university [14]. Four of the articles were designed to evaluate and assess healthcare professionals' knowledge of pain management and included the following: 37 registered nurses working in the postanesthesia care unit at a department of anesthesiology and critical care medicine in a university hospital in Baltimore [15]; 40 registered nurses (22 nurses from an academic medical center and 18 nurses from a community-based regional medical center) and the electronic health records of 58 patients from an academic medical center [16]; 23 nurses employed in a trauma unit in an urban academic hospital in Western Pennsylvania [17]; and 51 nurses working at three medical and surgical units at a university hospital in Indiana [1]. The results are presented in relation to the two research questions.

### 3.1. PMEP Impact on Surgical Nurses Leading to Successful Pain Outcomes for Patients

Multiple forms of technology-based training were utilized in those studies that found PMEP to be effective, and these education programs consisted of realistic simulated scenarios and video scenarios to engage both students and qualified nurse participants to apply knowledge to make clinical decisions. The advantage of the computer-based simulation tools was that they illustrated the effects of the chosen invention and provided guidance on optimal pain management techniques. In addition, the technology specifically led to improving nurses' abilities to change their practice in relation to the most appropriate pain management as the scenarios were based on realistic clinical situations. The PMEPs included tasks relating to the assessment of pain within the domains of pain knowledge, assessment, pharmacology, and interventions [1]. According to Keen and colleagues [1], by using electronic teaching methods, PMEPs can promote learning about evidence-based practice while being offered within cost-effective pedagogies that are sustainable and can be spread across large educational facilities. The advantage of this educational program is that not only does it influence nursing knowledge and attitudes but it also addresses the complexities of pain management in nursing. The computer-based simulation teaching tool was found to influence students' perceptions of learning pain management skills, who found them to be more effective than traditional lectures, with the potential to change nursing practice [1, 14]. However, differences within nursing practice can also affect the success of a patient's pain outcomes. For example, the study conducted by Eaton and colleagues [16] found that variations among nurses' practices influenced how they implemented evidence-based pain management (EBPM), i.e., years of nursing practice and level of nursing degree, as indicated by nurse documentation gathered from the electronic health record (EHR). They concluded that those involved in promoting an organization's EBP environment should be involved in establishing a standard quality of care, including national recognition as a “magnet designation,” as indicated by the American Nurses Credentialing Centre's Magnet Recognition Program; facilitation of best pain practices; measurement of success; fiscal commitment of human resources and technology; and the evaluation of evidence-based practice [16].

A major feature of the PMEPs [15] in this review was an ability to improve nurses' knowledge about effective pain management practice. In an evaluation of an evidence-based PMEP [15], the intervention included the introduction of an evidence-based pain management algorithm for nurses to use in pain assessment, based on enhancing patients' satisfaction with their pain management. The value of the intervention was reflected in improved hospital outcomes, such as a decrease in length of stay, an increase in patients' satisfaction, and a decrease in the cost of treatment [17]. In the study performed by Naqib and colleagues [15], the effectiveness of the PMEP intervention was evaluated by comparing self-reported patient postanesthesia care unit (PACU) surveys gathered pre- and postintervention, which included such variables as time to pain relief, PACU length of stay, and patient satisfaction with pain management. The results showed a decrease in the proportion of patients with opioid tolerance who required more than 60 minutes to achieve adequate pain relief (from 32.7% preintervention (*n* = 82) to 21.3% postintervention (*n* = 87)). Additionally, postintervention, the PACU length of stay was decreased by 53 minutes, and prolongation of PACU stay as a result of uncontrolled pain for opioid-tolerant patients decreased from 45.2% to 25.7%. This suggests that completing the PMEP had a positive impact on nurses' pain management practices, improving both patient satisfaction and postoperative outcomes. Considering the effectiveness of the evidence-based pain education programs included in this review, many of the samples were not representative of the larger population of nurses due to the relatively small sample sizes. One study [1] reported that their results indicated a statistically significant difference in knowledge and attitudes about pain management when comparing the mean survey scores preintervention and postintervention scores of nurses' knowledge and attitudes. However, we were concerned about the large proportion of respondents who withdrew from the study between the two time points. In order to determine the strength of the results, we therefore calculated the effect size from the published results, comparing the mean preintervention and postintervention scores. We calculated Cohen's *d* “effect size” of the difference between the mean preintervention and postintervention scores by using the mean, number of values, and standard deviation. This confirmed that the intervention achieved only a medium effect size (*d* = 0.65).

### 3.2. Use of Mixed Methods to Study PMEP (from the Five Articles)

We found that a mixed-methods approach was utilized for each of the PMEP evaluations included in this review. Each had the aim of improving the quality of care in pain management, and each found that the PMEP interventions met this aim, as indicated by the results extracted from the studies. These results suggest that introducing PMEP education programs in nursing curricula will help improve the effectiveness and efficiency of developing strategies for managing patients' pain relief. These methods were clearly described and were appropriately matched to each study's research aim, adding to the credibility and reliability of the results. A variety of methods were used for collecting and analyzing the data, such as using both quantitative and qualitative analysis of survey results [14], using a cross-sectional design to analyze questionnaire, medical records, and interview data [16], as well as quantitative measuring of the effectiveness of the intervention by using a pre- and posttest design, using a survey before and after implementation [1, 6, 17].

The results from all of the five studies found that the PMEP had made a significant impact on how nursing education can enhance practice, particularly where an education program is designed to improve the knowledge and competencies of the qualified nurse, as well as other healthcare providers, in improving their skills in critical thinking, leadership, case management, and health promotion, and for their ability to practice across a variety of inpatient and outpatient settings, wherein nurses' practices involve managing patients' pain. Surprisingly, we found no available publications using a mixed-method design relating specifically to PMEPs for severe postoperative pain management in low- and middle-income countries, as the studies were limited to a wider clinical setting and only those based in the USA.

## 4. Discussion

The results show that PMEPs can enhance nursing practice with improved skills in critical thinking, leadership, patient care management, and health promotion. In all of the studies, the programs improved nurses' ability to practice across a variety of inpatient and outpatient settings, and nurses' practices in managing patient's pain improved after they completed the program. These results support our findings in previous studies, particularly in addressing issues relating to the “Responding to and addressing patients' postoperative pain system model,” with the overlapping of pain management systems [6]. According to our previous study results, we found that nurses assessed patients' pain by using double- and triple-control methods to document and record it, which created delays in treating and managing pain when providing pain relief [6]. We critiqued this model while taking into consideration the unnecessary complexity of the communication and information systems when providing pain relief to perioperative patients, which often caused delays in the recovery phases and in managing the patients' pain [6]. Our research also suggests ways in which nursing education can enhance practice by promoting person-centered pain management in surgical care. One conclusion that can be drawn from our research, in tandem with this review, is that, to address the theory-practice gap often referred to in nursing education, the curriculum should include more complex teaching methods when considering the function that the nurse has in the clinical environment. This review presents important considerations relating to nursing education and in enhancing their knowledge, skills, and attitudes relating to managing the patient in pain. We chose to use a scoping review method, which revealed a prevalence of the use of mixed methods in previous literature relating to the effectiveness of PMEP. This method is known to be a useful approach in the research of the complexity of the evaluation of education and practice in the nursing field [18]. Nevertheless, the very small number of articles identified is perhaps not surprising, given that all PMEPs conducted might not be reported.

The PMEPs included in the review presented a variety of teaching strategies for pain management practice, and these included the use of computer-based simulation, web-based facilitation, and video scenarios based on using an evidence-based algorithm in practice, with the aim of implementing nursing strategies to improve the quality of care and pain management outcomes in the surgical field [1, 14–17]. This is a fundamental element of nursing knowledge that is vital in preparing nurses' decision-making skills to allow them to provide adequate treatment in a surgical patient context.

One of the elements that acts as both a barrier and facilitator to the success of postoperative pain management practice is the attitudes of the nurses who care for the patient in pain after surgery. PMEPs offer a solution which can be as simple as refreshing their knowledge about effective pain management [1, 2]. However, the effectiveness of PMEP on patient outcomes remains a challenge in the developed world and in low- and middle-income countries, where the consequences of inadequately treated postoperative pain for the patient in pain after surgery is more of concern [2, 6, 9, 19]. The limitations of this review are related to its size, breadth, and the rigidity of the inclusion and exclusion criteria for PMEP definitions. Despite these limitations, this study provides a variety of possibilities for evaluating PMEP, which can be conducted by using mixed-methods research design approaches and by integrating the innovation of PMEP. Thus, PMEPs may provide facilitators for successful person-centered postoperative pain management by using a multifaceted intervention that engages with healthcare providers, focuses nurses on the needs of their patients, and, ultimately, improves patients' experiences of the health system as a whole.

## 5. Conclusion

The results show that PMEPs have used computer-based simulation, web-based facilitation, and video-based scenarios, drawing on an evidence-based approach as their educational points of departure. These results suggest that initiatives for providing these programs involve gathering many creative ideas together to develop PMEPs. Several limitations were identified through reviewing the reference lists of the initial 40 articles. Our review would make the following recommendations based on our findings and aimed to provide a review of diverse studies on the topic for future research developments. However, with these limitations in mind, this review still shows that it is important to consider PMEP for surgical nurses, as it has the potential to transform the ways in which both newly registered nurses and nursing students are trained in pain management skills so that they are prepared to work collaboratively and effectively with healthcare providers in a complex and evolving healthcare system in a variety of settings.

## Figures and Tables

**Table 1:** Results extracted from the included studies, i.e., the aim, intervention, key findings, and identified area of concern regarding the impact of a pain management education program (PMEP) for surgical nurses and the effectiveness of PMEP on patient outcomes (the charting of the data).

No.	Author	Year	Country	Intervention	Study design and method sample
1	Allred and Gerardi [14]	2017	USA	An interactive computer-based simulation training tool	A one-group, posttraining mixed-methods descriptive design, analyzing both quantitative and qualitative questionnaire survey responses of student nurses to evaluate a pilot pain management computer simulation training program. 30 participants.
2	Naqib et al. [15]	2018	USA	A multicomponent intervention including the introduction of a clinical pain pathway for multimodal analgesia for both opioid-naïve and opioid-tolerant patients undergoing surgery, and an educational program on pain management for frontline clinical nurses in a postanesthesia care unit	Quantitative measuring of the intervention's impact on time to pain relief, length of stay in unit, and patient satisfaction with pain management, as measured by self-report. PMEP comprised problem-based learning workshops using case scenario video recordings of patients' consultations with pain management specialists. 37 PACU nurse participants.
3	Eaton et al. [16]	2017	USA	An investigation of nurse-level and organizational-level factors associated with evidence-based cancer pain management practice using evidence gathered from nurses' documentation of pain management in patients' electronic health record, questionnaires, and interviews	A mixed-methods descriptive cross-sectional design, using questionnaires completed by 40 RNs, medical record abstraction and interviews with 12 RNs, 2 nurse managers, 3 clinical nurse specialists, 1 nurse educator, and 2 chief nursing officers (60 participants in total). Pain management documentation gathered from 58 patient records.
4	DeVore et al. [17]	2017	USA	A quality improvement intervention, including an in-service education program to introduce an evidence-based pain management algorithm and educate nurses on the manifestations, complications, and interventions relating to cancer pain management practice	A mixed-methods, descriptive evaluation design, using a pre- and posteducational test (6 items) and survey (3 items) during the in-service sessions. 23 nurse participants. Patients' satisfaction with their pain management was evaluated using documentation gathered from the hospital consumer assessment of healthcare providers and systems scores in the electronic records of 58 patients.
5	Keen et al. [1]	2017	USA	A targeted evidence-based pain education program consisting of two 30-minute interactive educational session one month apart	One-group, paired, completed a pre-test/post-test questionnaire survey (knowledge and attitudes survey regarding pain scale) to evaluate the effectiveness of the educational intervention. 51 nurses from medical and surgical inpatients units completed the pretest, 24 nurses completed both pre- and posttest surveys.

**Table 2:** Overviews of articles characteristic including design, year of publication, impact factor, number of participants, and country.

Study	1	2	3	4	5
Design	Quantitative^*∗∗*^ and qualitative^*∗∗∗*^	Contribution program and group discussion^*∗∗∗*^	Quantitative^*∗∗*^ and qualitative^*∗∗∗*^	Cross-sectional^*∗∗*^ survey with open-ended questions analyzed qualitatively^*∗∗∗*^	A survey method^*∗∗*^ to evaluate intervention
Year	2017	2018	2017	2017	2017
SJR impact factor	0.633	0.633	0.224	0.332	0.633
No. of participants	30 nursing students	37 RNs^*∗*^	60 RNs^*∗*^ 58 pts records	23 RNs^*∗*^	51 RNs^*∗*^
Country	USA	USA	USA	USA	USA

Notes: ^*∗*^policymakers, academics, and health professions (e.g., nurses and physicians), ^*∗∗*^questionnaire or list of statements, and ^*∗∗∗*^open-ended questions. RNs: registered nurses; pts: patients.

## Data Availability

The data used to support the findings of this study are available from the corresponding author upon request.
